# Personal space increases during the COVID-19 pandemic in response to real and virtual humans

**DOI:** 10.3389/fpsyg.2022.952998

**Published:** 2022-09-14

**Authors:** Daphne J. Holt, Sarah L. Zapetis, Baktash Babadi, Jordan Zimmerman, Roger B. H. Tootell

**Affiliations:** ^1^The Department of Psychiatry, Massachusetts General Hospital, Boston, MA, United States; ^2^Harvard Medical School, Boston, MA, United States; ^3^The Athinoula A. Martinos Center for Biomedical Imaging, Charlestown, MA, United States; ^4^The Department of Radiology, Massachusetts General Hospital, Boston, MA, United States

**Keywords:** personal space, social distancing, COVID-19, virtual reality, public health, anxiety

## Abstract

Personal space is the distance that people tend to maintain from others during daily life in a largely unconscious manner. For humans, personal space-related behaviors represent one form of non-verbal social communication, similar to facial expressions and eye contact. Given that the changes in social behavior and experiences that occurred during the COVID-19 pandemic, including “social distancing” and widespread social isolation, may have altered personal space preferences, we investigated this possibility in two independent samples. First, we compared the size of personal space measured before the onset of the pandemic to its size during the pandemic in separate groups of subjects. Personal space size was significantly larger in those assessed during (compared to those assessed before) the onset of the pandemic (all *d* > 0.613, all *p* < 0.007). In an additional cohort, we measured personal space size, and discomfort in response to intrusions into personal space, longitudinally before and during the pandemic, using both conventional and virtual reality-based techniques. Within these subjects, we found that measurements of personal space size with respect to real versus virtual humans were significantly correlated with one another (*r* = 0.625–0.958) and similar in magnitude. Moreover, the size of personal space, as well as levels of discomfort during personal space intrusions, increased significantly during (compared to before) the COVID-19 pandemic in response to both real and virtual humans (all *d* > 0.842, all *p* < 0.01). Lastly, we found that the practice of social distancing and perceived (but not actual) risk of being infected with COVID-19 were linked to this personal space enlargement during the pandemic (all *p* < 0.038). Taken together, these findings suggest that personal space boundaries expanded during the COVID-19 pandemic independent of actual infection risk level. As the day-to-day effects of the pandemic subside, personal space preferences may provide one index of recovery from the psychological effects of this crisis.

## Introduction

Personal space is the “comfort zone” surrounding the body that is typically maintained free of intrusions from others in order to protect the organism from harm (Hayduk, 1983; Graziano and Cooke, 2006). The monitoring and defense of this space is an evolutionarily conserved function of the brain across many species, from insects to mammals (Graziano and Cooke, 2006). In humans, the dimensions of personal space are moderately influenced by a number of situational, social, and psychological factors, including gender, age, social status, cultural norms, and psychological characteristics (Hayduk, 1983; Uzzell and Horne, 2006; Kennedy and Adolphs, 2014; Holt et al., 2015; Iachini et al., 2016). However, when many of these situational factors are controlled within a laboratory setting, the preferred distance that a given individual maintains from others remains remarkably stable over repeated measurements (Hayduk, 1981; Tootell et al., 2021).

Since early 2020, “social distancing” recommendations aiming to reduce transmission of the COVID-19 virus have influenced how far people stand from each other in many public settings. These consciously adopted distances (usually 6 feet in the US, and 2 meters elsewhere) are much larger than those generated by the intrinsic brain mechanisms involved in personal space regulation (e.g., 50–100 cm) (di Pellegrino and Làdavas, 2015). However, it is unclear whether the practice of social distancing, and other effects of the pandemic on social interactions (Killgore et al., 2020; Tull et al., 2020; Calbi et al., 2021), have broadly influenced personal space regulation. To examine this question, we measured personal space in two independent cohorts of subjects. In addition, in the second cohort, personal space size was measured with respect to both real people and avatars presented using virtual reality technology. With these data, we tested the prediction that the size of personal space, assessed in the laboratory using the well-validated Stop Distance Procedure (Hayduk, 1983; Kaitz et al., 2004), increased during the pandemic, even in a virus-free, virtual reality context.

## Materials and methods

### Participants

#### Cohort 1

A subset of the participants of a study of the mental health of college students (Burke et al., 2019; DeTore et al., 2022) underwent a comprehensive in-person clinical and cognitive assessment that included measurements of personal space size with human confederates (see details below). A total of 249 participants were assessed (65.1% female, mean age: 19.0), including (1) *n* = 178 in 2017–2019 (65.2% female; mean age: 19.0), (2) *n* = 38 in January and February of 2020, immediately prior to the beginning of the pandemic and the institution of the associated restrictions and mandates in Boston (68.4% female, mean age: 18.7), and (3) *n* = 33 after March 2020, during the pandemic (60.6% female, mean age: 19.3). There were no significant differences in age or gender across these three groups (see Supplementary Table S1 for additional demographic information about this cohort). The three groups were 100% independent of each other (with no common subjects). Also, the experimental procedures were identical across these groups, other than some additional precautions implemented during the pandemic (see below).

#### Cohort 2

A second cohort of healthy individuals (*n* = 19, 47% female, mean age: 30.6 ± 11.3 years) were recruited *via* online advertisement posted on the Massachusetts General Hospital (MGH) Rally Website^1^ and initially assessed before the COVID-19 pandemic lockdown began in Boston, MA (during the period between September 2019 and early March 2020; the pandemic lockdown in Boston began on March 13, 2020). A subset of this same group of subjects (*n* = 12, 42% female, mean age: 33.3 ± 11.2 years) returned to complete a second assessment session, which was identical to the first (other than the addition of pandemic-related precautions, see below), during the initial surge of the COVID-19 pandemic in Boston (July–December 2020; see Supplementary Table S2 for additional demographic information about this cohort). All subjects of the baseline sample who were willing and able to return were enrolled in the second session. The two sessions were an average of 10.04 ± 1.6 months apart. Intrinsic personal space preferences have been shown to be stable and measured reliably over that length of time (Hayduk, 1983).

All research protocols were approved by the Mass General Brigham Healthcare Institutional Review Board. Written informed consent was obtained from all subjects prior to enrollment.

### Overview of procedures

Throughout this study, we used a well-validated, highly reliable (kappa ~0.8) experimental procedure for measuring personal space size, the Stop Distance Procedure (SDP) (Hayduk, 1983; Kaitz et al., 2004). The SDP measures the distance from a subject at which the subject first becomes uncomfortable when another person (the experimental confederate) approaches them (passive trials), or when the subject approaches another person (active trials). Both types of trials measure the distance between the subject’s body and their personal space boundary.

To control additional variables that could potentially influence personal space size (such as varying physical characteristics of the SDP confederates), in Cohort 2 we also collected personal space measurements using an immersive virtual reality (VR) version of the SDP, in addition to the conventional SDP. This VR procedure measures personal space in response to virtual simulations of humans (“avatars”) but is otherwise identical to the SDP conducted with real humans. VR-based measurements of personal space with respect to avatars have been shown to correspond closely to those measured to real humans *in vivo* (Iachini et al., 2016; Tootell et al., 2021).

In addition, in Cohort 2, arousal responses to personal space intrusions (as reflected by subjective discomfort ratings) were measured at different distances within (as well as outside of) personal space boundaries, to both real and virtual humans (see details below).

### The conventional SDP

Passive SDP trials: Subjects were first asked to stand still while facing a human confederate (a laboratory staff member) who was standing 3 meters away from the subject. Subjects were instructed to maintain eye contact with the confederate, who maintained a neutral facial expression, and told that the confederate would start walking slowly toward them, and that they should say “okay” when the confederate reached the distance that the subject would typically maintain from a person they had just met. For these passive trials, the confederates were trained to walk at approximately 0.1 m/s. Passive SDP trials were collected in both Cohorts 1 and 2.

Active SDP trials: In Cohort 2, the active version of the SDP was also conducted, in addition to the passive version. Active trials began similarly to the passive trials, with the subject standing 3 meters away from the confederate. However, in the active version of the procedure, the subjects were instructed to approach the confederate, and to stop at the distance described above and say “okay.” Again, subjects were asked to maintain eye contact with the confederate, who maintained a neutral facial expression.

Both the active and passive SDP trials were conducted with a male and a female confederate, in a counterbalanced order, with two trials per gender.

### The VR-based SDP

A HTC VIVE Virtual Reality System was used to collect the VR-based SDP and the measurements of responses to personal space intrusions by avatars. A head-mounted display (HMD) presented stereoscopic images at a resolution of 1,080 × 1,200 pixels per eye, with a 110° field of view at a refresh rate of 90 Hz. A software program for measuring personal space (designed by the research team and developed by Productive Edge,^2^ Chicago, Illinois, United States) was run *via* a SteamVR platform on an Alienware 15 R3 Laptop. In the HMD, each avatar was presented in the identical simple environment (a room with white walls, see Supplementary Figure S1). The avatars (i.e., non-player characters) could be placed at different distances from the subjects and could appear to walk toward subjects while maintaining eye contact with them. Both active and passive SDP trials were conducted using four different avatars (two males and two females, 50% non-white in appearance). The SDP type (active or passive), SDP modality (real or virtual), and confederate order were counterbalanced across subjects. There were two trials per avatar, with a total of eight active trials and eight passive trials (16 trials per time point).

In the VR environment, the height of each avatar was set to equal the height of the subject, and the approach speed was set at 0.1 m/s. As with the conventional SDP, in the passive trials, subjects were asked to stand still and maintain eye contact with an avatar that began walking toward them. The subjects were instructed to say “okay” when the avatar reached the distance that they would typically maintain from such a person they had just met. During the active trials, the subjects were instructed to approach the avatar, and to stop at this distance and at the same time say “okay.”

### Summary of design and number of trials of the SDP

Thus, for Cohort 1, the SDP included only passive trials with human confederates (the standard procedure), with a total of 4 trials collected per subject (two trials per each confederate gender).

In Cohort 2, the SDP included both passive and active trials (with two trials per each confederate gender), with both human (1 male and 1 female: 4 trials × 2 (passive and active) = 8 trials) and avatar (2 male and 2 female: 8 trials × 2 (passive and active) = 16 trials) confederates (a total of 24 SDP trials), at two time points (before and during the pandemic). Thus for Cohort 2, a total of 48 SDP trials were collected per subject.

### Responses to personal space intrusions

In Cohort 2, discomfort in response to personal space intrusions was also measured, in addition to personal space size. First, personal space size was calculated independently in each individual subject for each of the two SDP modalities (real and virtual), using the average personal space size measured in the active trials of that visit, which are slightly more stable than the passive trials (Tootell et al., 2021). Then multiples of each individual subject’s personal space size (25, 50, 100, 200, and 400%) were calculated. To measure discomfort in response to personal space intrusions, real or virtual humans were presented in separate runs at these 5 distances from the subject in a counterbalanced, pseudorandomized order. For each trial, the subject began the trial with their eyes closed, and then was asked to open their eyes during the presentation of each stimulus. The subject was instructed to stand still during the stimulus presentation and maintain eye contact with the real or virtual human. During each presentation, subjects were asked to rate their agreement to the statement “I want to move away” (indicating subjective discomfort) on a Likert scale from 1–5 (1: not at all, 3: somewhat, 5: very much). The order of modality (i.e., of the two procedures conducted with real vs. virtual humans) was the same as the order used for the initial SDP measurement within each subject and visit (Tootell et al., 2021).

### Fitting power law functions

A prior study demonstrated that relative magnitudes of discomfort in response to varying personal space intrusions (as above) were best approximated by a power law function (Tootell et al., 2021). To test whether such a pattern of discomfort responses was altered during the pandemic in Cohort 2, power law functions as $D=a\;x^{b}$ were fitted to the pooled discomfort ratings for each time point, where D is the reported discomfort level, x is the distance between the subject and the real or virtual human (as a percentage of pre-pandemic personal space size), and a,b (the prefactor and the exponent, respectively) are parameters obtained by minimizing the sum squared error between the power law function and the data. Separate power law functions were fitted to the data collected before and during the pandemic, and for the procedures using real and virtual humans. To test whether the power law functions were significantly different before versus during the pandemic, the fitting procedure was repeated 1,000 times in each case, by bootstrapping the data with substitution. The resultant a and b parameters of the two time points (before and during the pandemic) were compared using the nonparametric two-sample Kolmogorov–Smirnov (KS) test, separately for real and virtual humans.

### COVID-19 safety procedures

For assessments occurring during the pandemic, subjects were screened for COVID-19 symptoms and travel within 48 h of arrival in accordance with MGH guidelines. In addition, mask-wearing and social distancing policies were in effect for all subjects and staff members throughout the majority of the research visits. The only exception to this (approved by the MGH COVID safety team) was during the SDP measurement of personal space to real humans; in this case, the subject wore a mask and protective eye goggles, while the confederate (i.e., staff member) did not wear a mask. This was done in order to maintain the same SDP conditions from the perspective of the subject (facing someone who is not wearing a mask) before and during the pandemic. Immediately following the SDP procedure, the staff member resumed wearing a mask.

### Statistical analyses

#### Cohort 1

A one-way ANOVA was used to test for differences among the three groups in the size of personal space, and significant effects were followed up by Independent Sample *t*-tests, to test the hypothesis that personal space was larger during, compared to before, the pandemic.

#### Cohort 2

Repeated-measure ANOVAs (modality × time) and paired samples *t*-tests were used to test for differences in personal space size and discomfort ratings across modality (real and virtual) and the two time points (before and during the pandemic), to test the hypothesis that personal space size and discomfort in response to personal space intrusions increased during vs. before the pandemic in this cohort. Significance values (for paired *t*-tests comparing discomfort ratings across distances) were corrected for multiple comparisons (alpha = 0.05, Bonferroni corrected), within each time point and modality. Change scores were calculated as the difference between values collected at the second and first time point (i.e., “During” minus “Before” the COVID-19 pandemic). Thus, a positive change score indicated an increase in the respective measure over time.

#### Correlations

Because some of the Cohort 2 personal space measurements and the self-report questionnaire data were not normally distributed, Spearman’s correlations were used in the correlation analyses, including those measuring relationships between (1) personal space size during the pandemic and (2) changes in personal space size over time and:

1. local rates of COVID-19 cases, measured as the positive COVID-19 case rate during the previous 2 weeks in the town in which the subject lived (obtained from Massachusetts Department of Public Health COVID-19 data archive).^3^
2. self-reported beliefs and experiences related to the pandemic (Gerhold, 2020), including the perceived risk of COVID infection and the practice of social distancing.

Correlations with symptoms of psychopathology and distress were also explored, including anxiety and distress related to the pandemic, as well as levels of depression (Beck et al., 1961), anxiety (Spielberger et al., 1983), and subclinical psychotic symptoms (Peters et al., 1999; Supplementary Table S1).

## Results

### Cohort 1

**Figure 1:**
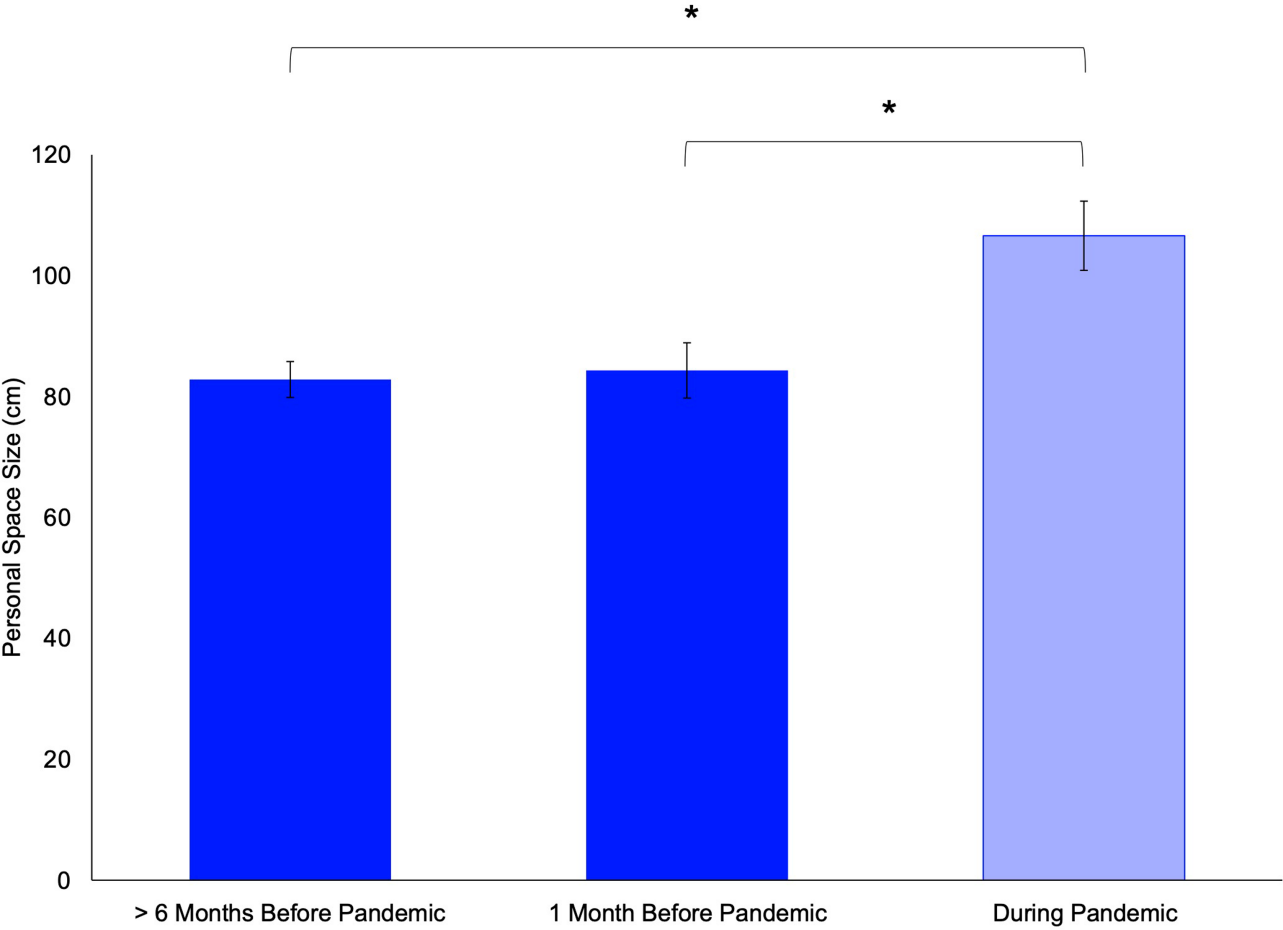
The size of personal space was larger during (compared to before) the pandemic (Cohort 1). Bar plots of mean personal space size, as measured by the standard Stop Distance Procedure (using human confederates), of the three groups of subjects in Cohort 1 are shown. Personal space size was significantly larger in the group assessed during the pandemic (light blue bar) compared to (1) those who had been assessed in early 2020 [1 month before the pandemic; *t*(69) = −3.076, *p* = 0.003; right dark blue bar] and (2) those who had been assessed well before the pandemic [> 6 months before the pandemic; *t*(209) = −3.238, *p* = 0.001; left dark blue bar]. There was no significant difference between the mean personal space size of the two groups assessed before the pandemic [*t*(214) = −0.222, *p* = 0.824]. Error bars indicate standard errors of the mean. ^*^ *p* < 0.005.

A one-way ANOVA [*F*(246,248) = 5.698, *p* = 0.004] revealed that in Cohort 1, the size of personal space (measured with respect to real humans) was significantly larger in the group assessed during the pandemic compared to both: (1) those assessed in early 2020 [*t*(69) = −3.076, *p* = 0.003] and (2) those assessed more than 6 months before the pandemic [*t*(209) = −3.238, *p* < 0.001; Figure 1].

### Cohort 2

As expected (Tootell et al., 2021), in Cohort 2, the size of personal space with respect to real humans was highly correlated with the size of personal space to virtual humans (avatars) across individuals, for both the passive and active trials, both before [passive trials: *r*(17) = 0.625, *p* = 0.004; active trials: *r*(17) = 0.644, *p* = 0.003] and during [passive trials: *r*(10) = 0.958, *p* < 0.001; active trials: *r*(10) = 0.790, *p* = 0.002] the COVID-19 pandemic.

**Figure 2:**
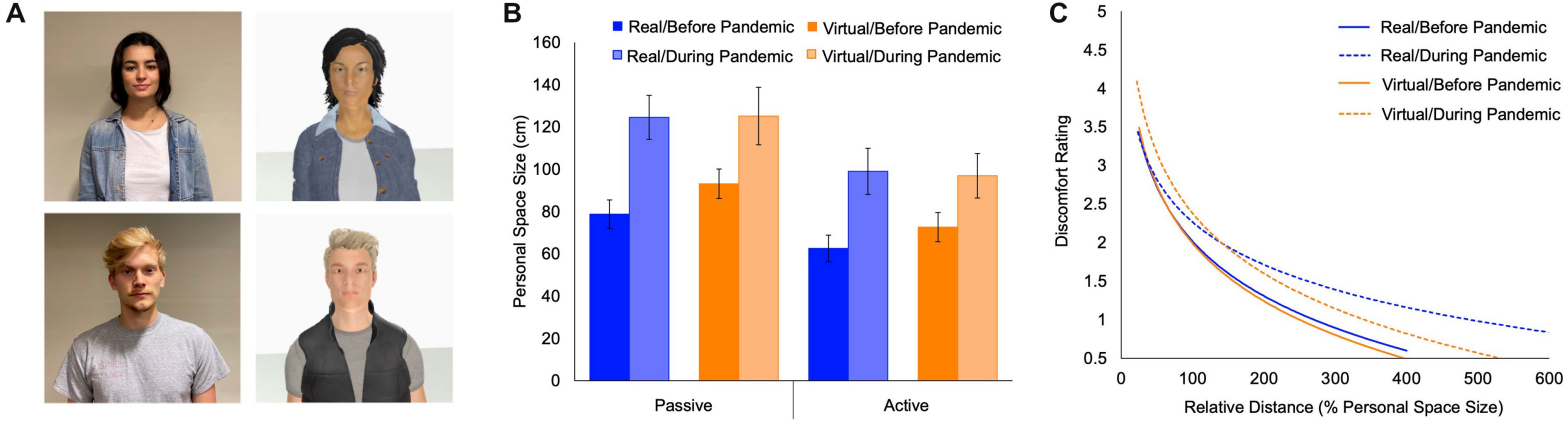
Personal space size and discomfort during personal space intrusions increased longitudinally within individuals during the COVID-19 pandemic (Cohort 2). **(A)** Examples of real and virtual human confederates that were used in the Stop Distance Procedure (SDP) and **(B)** the measurements of mean personal space size in Cohort 2 are shown. Personal space size, measured using the SDP with real and virtual humans, increased significantly during the COVID-19 pandemic within individuals. Also, the increases in personal space during the pandemic to the real and virtual humans correlated with each other (all *r* > .608; all *p* < .036). **(C)** Power law fits to the before- and during-pandemic discomfort ratings, as a function of distance from real or virtual humans, expressed as percentages of before-pandemic personal space size, are shown.

In addition, within these Cohort 2 subjects, the size of personal space was significantly larger during, compared to before, the COVID-19 pandemic for all four measurements of personal space size [real humans: passive trials: *t*(11) = 5.732, *d* = 1.655, *p* < 0.001; active trials: *t*(11) = 3.863, *d* = 1.115, *p* = 0.003; virtual humans: passive trials: *t*(11) = 2.918, *d* = 0.842, *p* = 0.014; active trials: *t*(11) = 3.082, *d* = 0.890, *p* = 0.01; see Table 1; Figure 2; Supplementary Table S3]. Also, these changes in personal space size during the pandemic to real and virtual humans were significantly correlated with each other (passive trials: *r* = 0.608, *p* = 0.036; active trials: *r* = 0.762, *p* = 0.004; See Supplementary Figure S2).

**Table 1:** Personal space size measurements, Cohort 2.

	Personal space size to real humans				Personal space size to virtual humans			
	Baseline	Before the pandemic	During the pandemic	Percent change	Baseline	Before the pandemic	During the pandemic	Percent change
Passive trials	82.6 (26.9)	78.8 (23.3)	124.5 (36.0)	58.0%	91.67 (26.3)	93.1 (24.1)	125.1 (47.1)	34.4%
Active trials	67.3 (25.2)	62.0 (21.7)	99.0 (37.8)	59.7%	73.7 (25.1)	72.6 (23.9)	96.9 (36.5)	33.5%

Mean personal space size in centimeters [mean (standard deviation)] and percentage change in personal space size, measured with respect to real and virtual humans, in Cohort 2 at baseline (*n* = 19) and in those who were assessed at the two time points (before and during the pandemic, *n* = 12). There were no significant differences between the Baseline (*n* = 19) and Before the Pandemic (*n* = 12) means (all *p* > 0.59).

Prior work has shown that intrusions into personal space by unfamiliar others lead to an increase in discomfort at progressively closer distances (Felipe and Sommer, 1966; Hayduk, 1981; Llobera et al., 2010; Schoretsanitis et al., 2016), perhaps following a power law function (Tootell et al., 2021). To test whether such personal space intrusion-driven discomfort levels changed during the pandemic, subjects were asked to rate their discomfort in response to real and virtual humans, which were presented at a range of distances (25, 50, 100, 200, 400% of each subject’s personal space size, see “Materials and methods”), both before and during the pandemic.

The discomfort levels as a function of distance followed a power law fall-off, as previously (Tootell et al., 2021) in all four cases (to real humans, before and during the pandemic, respectively: $R^{2}=0.71$ and 0.67 ; to virtual humans, before and during the pandemic respectively, $R^{2}=0.73$ and 0.74 ). During the pandemic, discomfort to personal space intrusions increased significantly compared to the pre-pandemic discomfort ratings in response to both real and virtual humans, following a power law (real humans: *p* < 0.0001, KS statistic 0.53; virtual humans: *p* < 0.0001; KS statistic 0.21; Figure 2C). Specifically, the prefactor a was significantly different between the two timepoints (*p* < 0.0001 for both real and virtual humans, KS statistics 0.36 and 0.20, respectively) and the exponent b was significantly different between the two timepoints (*p* < 0.0001 for both real and virtual humans, KS statistics 0.53 and 0.21, respectively).

### Correlations with beliefs and experiences during the pandemic

**Figure 3:**
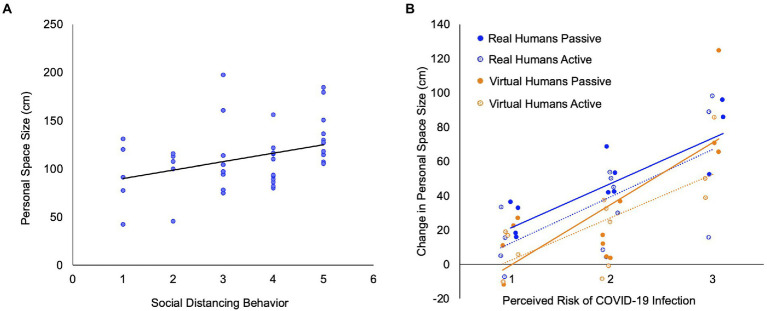
Associations with social distancing behavior and perceived risk of being infected with COVID-19 during the pandemic. **(A)** There was a significant correlation between personal space size during the pandemic and social distancing behavior (as assessed using ratings of the statement “I stay at least 6 feet away from people when I am outside”; *r*(41) = 0.358, *p* = 0.019) in the subjects assessed during the pandemic (31 subjects of Cohort 1 and 12 subjects of Cohort 2; total *n* = 43). Two subjects of Cohort 1 did not complete the scale measuring beliefs and experiences related to the pandemic. **(B)** Across all four personal space measurements (i.e., real and virtual, passive and active SDP trials), the change in personal space size observed in Cohort 2 that occurred following the onset of the pandemic (During – Before) was significantly positively correlated with perceived risk of COVID-19 infection (Real: passive: *r*(10) = 0.745, *p* = 0.005, active: *r*(10) = 0.656, *p* = 0.021; Virtual: passive: *r*(10) = 0.603, *p* = 0.038, active: *r*(10) = 0.738, *p* = 0.006).

For those assessed during the pandemic (of Cohorts 1 and 2 combined, *n* = 43), personal space size (in response to real humans, passive trials) was significantly positively correlated with social distancing behavior (ratings of “I stay at least 6 feet away from people when I am outside”; *r*(41) = 0.358, *p* = 0.019; Figure 3A). There were no significant correlations between personal space size during the pandemic and perceived or actual risk of infection, COVID-related anxiety or distress or any psychopathology measure (all *p* > 0.126).

In Cohort 2, the within-subject increase in personal space size during the pandemic in response to both real and virtual humans was significantly correlated with the *perceived* risk of being infected with the COVID-19 virus (ratings of “How likely do you think it is that you might become infected with COVID-19 in the near future?”) across all four personal space measurements (all *r* > 0.603; all *p* < 0.038; Figure 3B). In contrast, there were no correlations between the increase in personal space size during the pandemic and rates of *actual* infection, as reflected by case rates in the towns where the subjects lived. Perceived and actual risks of COVID infection were not correlated with each other (*r* = −0.030, *p* = 0.927).

Also, ratings of pandemic-related anxiety and distress and social distancing behaviors during the pandemic did not correlate with the increase in personal space size during the pandemic (all *p* > 0.073).

## Discussion

### Summary of findings

Here we report evidence derived from two independent cohorts of subjects that personal space boundaries expanded during the COVID-19 pandemic. In the first cohort, an increase in personal space size was observed in individuals assessed during the pandemic in comparison to two similar groups assessed either immediately before, or greater than 6 months before, the beginning of the pandemic. In a second smaller cohort, comprehensive measurements of personal space characteristics, collected both before and during the pandemic in the same subjects, revealed a large (~40–50%) increase in personal space size following the onset of the pandemic, accompanied by an increase in discomfort with the physical proximity of others. These longitudinal changes in personal space size occurred in response to both real humans and to avatars encountered in a virtual setting in the absence of COVID infection risk. The fact that the identical effect was observed in response to both real humans and avatars suggests that changes in personal space regulation during the pandemic became somewhat habitual and automatic over time.

Consistent with this interpretation, we also found that the size of personal space during the pandemic was significantly correlated with social distancing. Prior evidence for plasticity in the intrinsic mechanisms involved in monitoring external space near the body (Canzoneri et al., 2013; Martel et al., 2016; Serino, 2019) suggests that such plasticity occurring in response to social distancing or isolation may underlie changes in personal space-related behaviors during the pandemic. Thus, the current data raise the possibility that experience-dependent modifications in personal space regulation can be maintained and reinforced over time by a habitual behavior such as social distancing. However, further testing of this hypothesis is necessary to fully understand the mechanisms underlying these behavioral changes.

In addition, the perceived, but not the actual, risk of being infected with COVID-19 was correlated with the pandemic-associated change in personal space size in the second cohort. Thus, beliefs about the infectiousness of the virus may have contributed to a preference for greater distance from others during the pandemic, which was manifested even in response to avatars encountered in an immersive virtual reality environment in this study. This link between personal space size and perceived risk of infection replicates and extends a prior finding of an association between self-reported personal space preferences (assessed using a projective, online scale) and perceived, but not actual, COVID-19 infection risk during the early pandemic (Iachini et al., 2021). It is also consistent with a finding of an association between greater segregation of near and far space during the pandemic and greater germ aversion (Serino et al., 2021).

Intriguingly, interpersonal distances measured during the pandemic have been found to be smaller if the confederate in projective measurements of such distances appears to be wearing a mask when compared to non-mask-wearing confederates (Cartaud et al., 2018; Lisi et al., 2021; Biggio et al., 2022). These findings suggest that the presence of a mask elicits a sense of safety that influences personal space regulation. Based on these findings, we can speculate that the inclusion of mask-wearing confederates in the current study might have reduced or eliminated the pandemic-linked increases in personal space size. However, given that we found that perceived risk of COVID infection was not correlated with actual risk, and perceived infection risk was associated with increases in personal space size during the pandemic, it is possible that the presence of masks (and knowledge about their protective effects) would not have strongly impacted these results.

Subjective discomfort ratings increased in concert with the observed increases in personal space size in the current study. These findings are broadly consistent with other evidence for discomfort with the physical proximity of others during the pandemic, such as higher arousal ratings and more negative appraisals of images depicting large social gatherings during the early pandemic (Massaccesi et al., 2021). The time course of this discomfort response (i.e., the length of time it may take to abate after the most threatening aspects of the pandemic, related to the risks for serious illness, death, or loss, have substantially lessened) remains unclear.

### The functions of personal space

Although one goal of maintaining a safety zone around the body is the avoidance of harm (Graziano and Cooke, 2006), in humans there are clearly other functions of personal space-related behaviors beyond the physical protection of the body. Adjustments in personal space during social interactions are used by humans to communicate non-verbal, social signals (Hayduk, 1983) similar to the way that other forms of “body language” convey this type of information to others. For example, smaller interpersonal distances can signal trust, support, or comfort, whereas larger distances can convey fear or respect. During the pandemic, this normally automatic channel of social information exchange has not been fully available in many circumstances, i.e., it has been blunted or modified in many contexts due to social distancing practices, concerns about infection risk, and related avoidance behaviors. The specific impediment to social communication associated with the blunting of “natural” personal space regulation during the pandemic is analogous to that associated with wearing masks (i.e., mask-related interference with facial affect recognition; Pavlova and Sokolov, 2022). Given the length of time that such practices were in effect (and are still intermittently reinstated or voluntarily adopted) in some parts of the world, it is not surprising that this specific form of nonverbal communication may have been impacted. Some individuals may require time to regain full use of some of these tools of social interaction, such as personal space regulation.

In addition, individuals who had experienced some impairments in these domains or who had not yet fully developed these skills (e.g., children) before the pandemic may find this period of recovery (or transition to an endemic phase of the pandemic) particularly challenging. Personal space abnormalities have been observed in autism (Kennedy and Adolphs, 2014; Asada et al., 2016), schizophrenia (Park et al., 2009; Holt et al., 2015; Schoretsanitis et al., 2016; Lee et al., 2021; Zapetis et al., 2022), and Post Traumatic Stress Disorder (Bogović et al., 2016) and have been linked to loneliness (Layden et al., 2018), anxiety (Iachini et al., 2015) and social functioning impairments (Nechamkin et al., 2003; Holt et al., 2015; Zapetis et al., 2022). Thus, persistently impaired regulation of personal space in certain individuals could indicate a need for further evaluation, close monitoring or therapeutic intervention.

### The neural basis of changes in personal space during the pandemic

Although personal space-related behaviors have been linked to the function of the network of parietal and frontal cortical brain regions involved in monitoring the space near the body (Graziano and Cooke, 2006; Huang et al., 2012; Cléry et al., 2015; di Pellegrino and Làdavas, 2015), it is not known whether the function or structure of this network has been altered in parallel with changes in personal space-related behaviors during the pandemic. Given that the functional connectivity of this network (Holt et al., 2014; Zapetis et al., 2022) and variability in its responses (Ferri et al., 2015) have been linked to individual differences in personal space preferences, it is possible that changes in this circuit may have accompanied habitual enlargements in personal space during the pandemic. If such changes are persistent, longitudinal neuroimaging studies may be able to detect them and potentially shed light on some of the mechanisms underlying the plasticity of personal space regulation.

### Limitations and future directions

The findings of this study must be interpreted with caution due to several limitations of this work. The sample size of the second cohort was small, and inclusion in the second assessment of this cohort was based on the subjects’ willingness and ability to participate in research during the pandemic. However, the effect sizes of the longitudinal changes observed in this cohort were consistently large across all four measurements of the size of personal space (0.84 to 1.66), suggesting that these findings are relatively robust. Follow-up studies will be necessary to determine the time course of these changes as society emerges from the pandemic and resumes social activity levels that are closer to pre-pandemic norms. For those with persistent fears about the risks associated with physical proximity to others, the development of behavioral interventions that address these concerns may be helpful.

## Data availability statement

The datasets presented in this study can be found in online repositories. The names of the repository/repositories and accession number(s) can be found at: https://osf.io/hp2n4/?view_only=a017443177bf425087daccd1ca86fd74.

## Ethics statement

The studies involving human participants were reviewed and approved by Massachusetts General Brigham Institutional Review Board. The participants provided their written informed consent to participate in this study. Written informed consent was obtained from the individual(s) for the publication of any potentially identifiable images or data included in this article.

## Author contributions

DH developed the study concept, obtained the funding for the project, and oversaw the study. DH, SZ, and RT were involved in the study design. SZ and JZ collected the data. SZ, JZ, and BB analyzed the data. DH and SZ drafted the manuscript. RT, BB, and JZ revised the manuscript. All authors contributed to the article and approved the submitted version.

## Funding

This work was supported by the Research Scholar Program of the Executive Committee on Research of Massachusetts General Hospital (DH), and the National Institute of Mental Health (5R01MH109562; DH), and the MGH Translational Neuroscience Training for Clinicians Program (T32MH112485; BB).

## Conflict of interest

The authors declare that the research was conducted in the absence of any commercial or financial relationships that could be construed as a potential conflict of interest.

## Publisher’s note

All claims expressed in this article are solely those of the authors and do not necessarily represent those of their affiliated organizations, or those of the publisher, the editors and the reviewers. Any product that may be evaluated in this article, or claim that may be made by its manufacturer, is not guaranteed or endorsed by the publisher.

## Footnotes

^1^ https://rally.massgeneralbrigham.org/

^2^ https://www.productiveedge.com/

^3^ https://www.mass.gov/info-details/archive-of-covid-19-cases-in-massachusetts

## References

[ref1] Asada K. Tojo Y. Osanai H. Saito A. Hasegawa T. Kumagaya S. (2016). Reduced personal space in individuals with autism Spectrum disorder. PLoS One 11:e0146306. doi: 10.1371/journal.pone.0146306, PMID: 26814479 PMC4729526

[ref2] Beck A. T. Ward C. H. Mendelson M. Mock J. Erbaugh J. (1961). An inventory for measuring depression. Arch. Gen. Psychiatry 4, 561–571. doi: 10.1001/archpsyc.1961.01710120031004 13688369

[ref3] Biggio M. Bisio A. Bruno V. Garbarini F. Bove M. (2022). Wearing a mask shapes interpersonal space during COVID-19 pandemic. Brain Sci. 12:682. doi: 10.3390/brainsci12050682, PMID: 35625068 PMC9139907

[ref4] Bogović A. Ivezić E. Filipčić I. (2016). Personal space of war veterans with PTSD: some characteristics and comparison with healthy individuals. Psychiatr. Danub. 28, 77–81. 26938826

[ref5] Burke A. S. Shapero B. G. Pelletier-Baldelli A. Deng W. Y. Nyer M. B. Leathem L.. (2019). Rationale, methods, feasibility, and preliminary outcomes of a Transdiagnostic prevention program for at-risk college students. Front. Psych. 10:1030. doi: 10.3389/fpsyt.2019.01030, PMID: 32158406 PMC7051934

[ref6] Calbi M. Langiulli N. Ferroni F. Montalti M. Kolesnikov A. Gallese V.. (2021). The consequences of COVID-19 on social interactions: an online study on face covering. Sci. Rep. 11:2601. doi: 10.1038/s41598-021-81780-w, PMID: 33510195 PMC7844002

[ref7] Canzoneri E. Ubaldi S. Rastelli V. Finisguerra A. Bassolino M. Serino A. (2013). Tool-use reshapes the boundaries of body and peripersonal space representations. Exp. Brain Res. 228, 25–42. doi: 10.1007/s00221-013-3532-2, PMID: 23640106

[ref8] Cartaud A. Ruggiero G. Ott L. Iachini T. Coello Y. (2018). Physiological response to facial expressions in Peripersonal space determines interpersonal distance in a social interaction context. Front. Psychol. 9:657. doi: 10.3389/fpsyg.2018.00657, PMID: 29867639 PMC5949865

[ref9] Cléry J. Guipponi O. Wardak C. Ben Hamed S. (2015). Neuronal bases of peripersonal and extrapersonal spaces, their plasticity and their dynamics: knowns and unknowns. Neuropsychologia 70, 313–326. doi: 10.1016/j.neuropsychologia.2014.10.022, PMID: 25447371

[ref10] DeTore N. R. Luther L. Deng W. Zimmerman J. Leathem L. Burke A. S.. (2022). Efficacy of a transdiagnostic, prevention-focused program for at-risk young adults: a waitlist-controlled trial. Psychol. Med. 1-10, 1–10. doi: 10.1017/S0033291722000046, PMID: 35227342 PMC9433469

[ref11] di Pellegrino G. Làdavas E. (2015). Peripersonal space in the brain. Neuropsychologia 66, 126–133. doi: 10.1016/j.neuropsychologia.2014.11.011 25448862

[ref12] Felipe N. J. Sommer R. (1966). Invasions of personal space. Soc. Probl. 14, 206–214. doi: 10.1525/sp.1966.14.2.03a00080

[ref13] Ferri F. Costantini M. Huang Z. Perrucci M. G. Ferretti A. Romani G. L.. (2015). Intertrial variability in the premotor cortex accounts for individual differences in Peripersonal space. J. Neurosci. 35, 16328–16339. doi: 10.1523/jneurosci.1696-15.2015, PMID: 26674860 PMC6605506

[ref14] Gerhold L. (2020). COVID-19: risk perception and coping strategies. *PsyArXi*. [Epub ahead of print]. doi: 10.31234/osf.io/xmpk4

[ref15] Graziano M. S. A. Cooke D. F. (2006). Parieto-frontal interactions, personal space, and defensive behavior. Neuropsychologia 44, 2621–2635. doi: 10.1016/j.neuropsychologia.2005.09.011, PMID: 17128446

[ref16] Hayduk L. A. (1981). The permeability of personal space. Can. J. Behav. Sci. 13, 274–287. doi: 10.1037/h0081182

[ref17] Hayduk L. A. (1983). Personal space: where we now stand. Psychol. Bull. 94, 293–335. doi: 10.1037/0033-2909.94.2.293

[ref18] Holt D. J. Boeke E. A. Coombs G. DeCross S. N. Cassidy B. S. Stufflebeam S.. (2015). Abnormalities in personal space and parietal–frontal function in schizophrenia. NeuroImage 9, 233–243. doi: 10.1016/j.nicl.2015.07.008, PMID: 26484048 PMC4573090

[ref19] Holt D. J. Cassidy B. S. Yue X. Rauch S. L. Boeke E. A. Nasr S.. (2014). Neural correlates of personal space intrusion. J. Neurosci. 34, 4123–4134. doi: 10.1523/JNEUROSCI.0686-13.2014, PMID: 24647934 PMC3960459

[ref003] Holt D. J. Zapetis S. L. Babadi B. Tootell R. B. H. (2021). Personal space Increases during the COVID-19 Pandemic in Response to Real and Virtual Humans. medRxiv. doi: 10.1101/2021.06.09.21258234 PMC9515568 36186356

[ref20] Huang R. S. Chen C. F. Tran A. T. Holstein K. L. Sereno M. I. (2012). Mapping multisensory parietal face and body areas in humans. Proc. Natl. Acad. Sci. U. S. A. 109, 18114–18119. doi: 10.1073/pnas.1207946109, PMID: 23071340 PMC3497759

[ref21] Iachini T. Coello Y. Frassinetti F. Senese V. P. Galante F. Ruggiero G. (2016). Peripersonal and interpersonal space in virtual and real environments: effects of gender and age. J. Environ. Psychol. 45, 154–164. doi: 10.1016/j.jenvp.2016.01.004

[ref22] Iachini T. Frassinetti F. Ruotolo F. Sbordone F. L. Ferrara A. Arioli M.. (2021). Social distance during the COVID-19 pandemic reflects perceived rather Than actual risk. Int. J. Environ. Res. Public Health 18:5504. doi: 10.3390/ijerph18115504, PMID: 34063754 PMC8196577

[ref23] Iachini T. Ruggiero G. Ruotolo F. Schiano di Cola A. Senese V. P. (2015). The influence of anxiety and personality factors on comfort and reachability space: a correlational study. Cogn. Process. 16, 255–258. doi: 10.1007/s10339-015-0717-6, PMID: 26232194

[ref24] Kaitz M. Bar-Haim Y. Lehrer M. Grossman E. (2004). Adult attachment style and interpersonal distance. Attach Hum. Dev. 6, 285–304. doi: 10.1080/14616730412331281520, PMID: 15513270

[ref25] Kennedy D. P. Adolphs R. (2014). Violations of personal space by individuals with autism spectrum disorder. PLoS One 9:e103369. doi: 10.1371/journal.pone.0103369, PMID: 25100326 PMC4123873

[ref26] Killgore W. D. S. Cloonan S. A. Taylor E. C. Dailey N. S. (2020). Loneliness: A signature mental health concern in the era of COVID-19. Psychiatry Res. 290:113117. doi: 10.1016/j.psychres.2020.113117, PMID: 32480121 PMC7255345

[ref27] Layden E. A. Cacioppo J. T. Cacioppo S. (2018). Loneliness predicts a preference for larger interpersonal distance within intimate space. PLoS One 13:e0203491. doi: 10.1371/journal.pone.0203491, PMID: 30188950 PMC6126853

[ref28] Lee H. S. Hong S. J. Baxter T. Scott J. Shenoy S. Buck L.. (2021). Altered Peripersonal space and the bodily self in schizophrenia: A virtual reality study. Schizophr. Bull. 47, 927–937. doi: 10.1093/schbul/sbab024, PMID: 33844019 PMC8266616

[ref29] Lisi M. P. Scattolin M. Fusaro M. Aglioti S. M. (2021). A Bayesian approach to reveal the key role of mask wearing in modulating projected interpersonal distance during the first COVID-19 outbreak. PLoS One 16:e0255598. doi: 10.1371/journal.pone.0255598, PMID: 34375361 PMC8354471

[ref30] Llobera J. Spanlang B. Ruffini G. Slater M. (2010). Proxemics with multiple dynamic characters in an immersive virtual environment. ACM Trans. Appl. Percept. 8, 1–12. doi: 10.1145/1857893.1857896

[ref31] Martel M. Cardinali L. Roy A. C. Farnè A. (2016). Tool-use: An open window into body representation and its plasticity. Cogn. Neuropsychol. 33, 82–101. doi: 10.1080/02643294.2016.1167678, PMID: 27315277 PMC4975077

[ref32] Massaccesi C. Chiappini E. Paracampo R. Korb S. (2021). Large gatherings? No, thank you. Devaluation of crowded social scenes During the COVID-19 pandemic. Front. Psychol. 12:689162. doi: 10.3389/fpsyg.2021.689162, PMID: 34135838 PMC8201791

[ref33] Nechamkin Y. Salganik I. Modai I. Ponizovsky A. M. (2003). Interpersonal distance in schizophrenic patients: relationship to negative syndrome. Int. J. Soc. Psychiatry 49, 166–174. doi: 10.1177/00207640030493002, PMID: 14626359

[ref34] Park S.-H. Ku J. Kim J.-J. Jang H. J. Kim S. Y. Kim S. H.. (2009). Increased personal space of patients with schizophrenia in a virtual social environment. Psychiatry Res. 169, 197–202. doi: 10.1016/j.psychres.2008.06.039, PMID: 19762087

[ref35] Pavlova M. A. Sokolov A. A. (2022). Reading covered faces. Cereb. Cortex 32, 249–265. doi: 10.1093/cercor/bhab311, PMID: 34521105

[ref36] Peters E. R. Joseph S. A. Garety P. A. (1999). Measurement of delusional ideation in the normal population: introducing the PDI (Peters et al. delusions inventory). Schizophr. Bull. 25, 553–576. doi: 10.1093/oxfordjournals.schbul.a033401, PMID: 10478789

[ref37] Schoretsanitis G. Kutynia A. Stegmayer K. Strik W. Walther S. (2016). Keep at bay!--abnormal personal space regulation as marker of paranoia in schizophrenia. Eur. Psychiatry 31, 1–7. doi: 10.1016/j.eurpsy.2015.10.001, PMID: 26655593

[ref38] Serino A. (2019). Peripersonal space (PPS) as a multisensory interface between the individual and the environment, defining the space of the self. Neurosci. Biobehav. Rev. 99, 138–159. doi: 10.1016/j.neubiorev.2019.01.016, PMID: 30685486

[ref001] Serino S. Trabanelli S. Jandus C. Fellrath J. Grivaz P. Paladino M. P.. (2021). Sharpening of peripersonal space during the COVID-19 pandemic. Curr. Biol. 31, R889–R890. doi: 10.1016/j.cub.2021.06.001, PMID: 34314711 PMC8188303

[ref39] Spielberger C. Gorsuch R. Lushene R. Vagg P. R. Jacobs G. (1983). Manual for the State-trait Anxiety Inventory. Palo Alto, CA: Consulting Psychologists.

[ref40] Tootell R. B. H. Zapetis S. L. Babadi B. Nasiriavanaki Z. Hughes D. E. Mueser K.. (2021). Psychological and physiological evidence for an initial ‘rough sketch’ calculation of personal space. Sci. Rep. 11:20960. doi: 10.1038/s41598-021-99578-1, PMID: 34697390 PMC8545955

[ref41] Tull M. T. Edmonds K. A. Scamaldo K. M. Richmond J. R. Rose J. P. Gratz K. L. (2020). Psychological outcomes associated with stay-at-home orders and the perceived impact of COVID-19 on daily life. Psychiatry Res. 289:113098. doi: 10.1016/j.psychres.2020.113098, PMID: 32434092 PMC7252159

[ref42] Uzzell D. Horne N. (2006). The influence of biological sex, sexuality and gender role on interpersonal distance. Br. J. Soc. Psychol. 45, 579–597. doi: 10.1348/014466605x58384, PMID: 16984722

[ref002] Zapetis S. L. Nasiriavanaki Z. Luther L. Holt D. J. (2022). Neural correlates of variation in personal space and social functioning in schizophrenia and healthy individuals. Schizophr Bull. doi: 10.1093/schbul/sbac052 PMC9434426 35661903

