# Supplementary material for: Personal space increases during the COVID-19 pandemic in response to real and virtual humans
Source: Front Psychol. 2022 Sep 14;13:952998. doi: 10.3389/fpsyg.2022.952998 (PMC9515568; doi:10.3389/fpsyg.2022.952998)
Supplement: Supplementary file 1 [file Data_Sheet_1.pdf]

***Supplementary Material* for "Personal Space Increases during the COVID-19 Pandemic in Response to Real and Virtual Humans"**

Supplementary Methods

Inclusion/Exclusion Criteria for Cohort 1: Because the ongoing parent study focuses on characterizing young adults who may be transdiagnostically at-risk for psychiatric illness later in life (Burke et al., 2019; DeCross et al., 2020; DeTore et al., 2022), inclusion criteria included: 1) age 18-23 years 2) a total score on the Beck Depression Inventory (Beck et al., 1961) > 5, or a total score on the Peters et al Delusions Inventory > 3 (Peters et al., 1999) and 3) no current psychiatric treatment (i.e., no treatment with psychotropic medications, other than medications used to treat Attention Deficit Disorder or related conditions, and no current psychotherapy).

Inclusion/Exclusion Criteria for Cohort 2: 1) age 18-55 years, 2) no psychiatric disorder, or active substance abuse during the previous 6 months (as assessed by the Mini International Neuropsychiatric Interview) or unstable medical condition (Sheehan et al., 1997). Also, subjects were screened for virtual reality (VR) sickness using the Simulator Sickness Questionnaire (Bruck and Watters, 2009), after spending approximately 10 minutes in the immersive VR system. No subjects were excluded due to VR sickness.

Cohort 1 and 2 differed from each other with respect to demographic and psychological characteristics (see below). However, the subgroups within each cohort assessed before vs. during the pandemic were matched on these characteristics (i.e., there were no significant differences across groups within the two cohorts).

Supplementarty Figures

(A) (B)

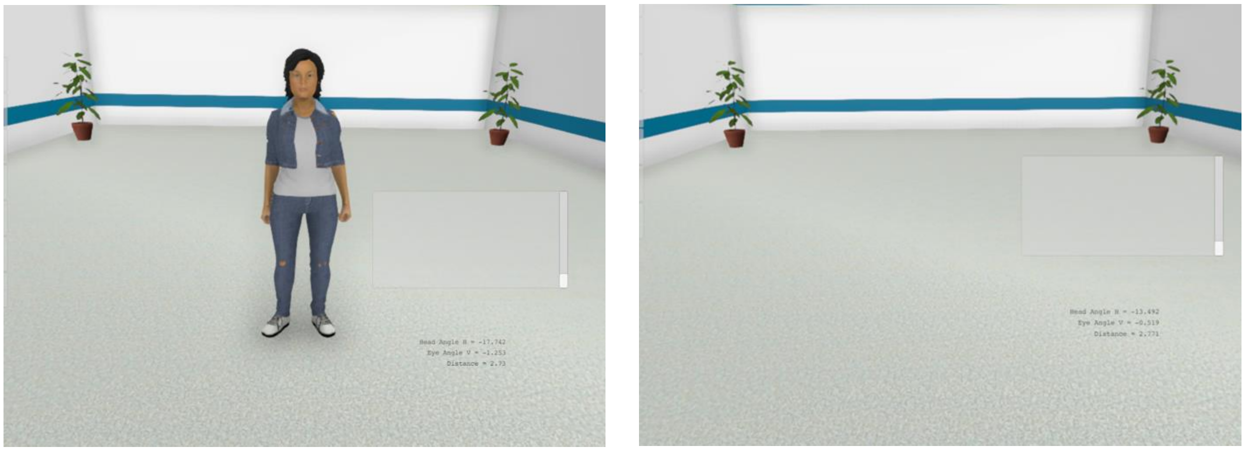

Figure S1. Images of the virtual environment in which the VR-based SDP was conducted. The fully immersive virtual environment consisted of a simple room with white walls; a plant in each corner and a blue stripe extending across the walls provided anchors for the perception of depth and physical scale. The room is shown with **(A)** and without **(B)** an avatar.

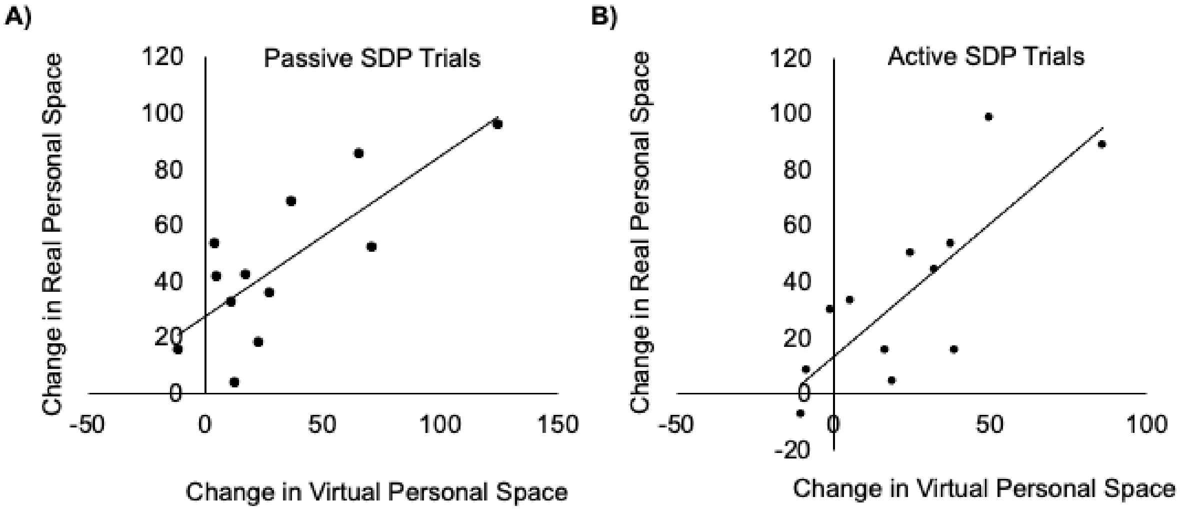

Figure S2. Correlations between personal space changes during the pandemic when measured with respect to real versus virtual humans. The increases in personal space during the pandemic to the real and virtual humans correlated with each other in both **(A)** passive (r = .608, p = .036) and **(B)** active (r = .762, p = .004) trials of the Stop Distance Procedure (SDP).

Supplementary Tables

	> 6 Months Before the Pandemic (n= 178)	1 Month Before the Pandemic (n= 38)	During the Pandemic (n= 33)
Age	19.0 (1.1)	18.7 (1.0)	19.3 (1.3)
Gender	65.2%	68.4%	60.6%
Ethnicity	20.2%	23.7%	15.2%
SES	15.9 (2.7)	16.3 (2.4)	15.3 (2.8)
Depression	11.3 (7.2)	13.0 (8.2)	14.6 (9.4)
State Anxiety	42.7(11.2)	45.2 (10.5)	47.1 (13.1)
Trait Anxiety	45.2 (10.7)	47.2 (10.6)	48.9 (11.1)
PEs	7.2 (5.7)	5.6 (3.4)	6.0 (3.5)

Table S1. Demographic and psychological characteristics of Cohort 1. Mean (mean (standard deviation)) age (in years), socioeconomic status (SES), psychopathology levels, as well as gender (% female) and ethnicity (% Hispanic) are shown for the three Cohort 1 groups. The racial composition of the groups was as follows: 1) > 6 Months Before the Pandemic: 57.8% Caucasian, 6.8% Black or African American, 31.1% Asian, 1.2% American Indian or Alaskan Native, and 3.1% declined to answer; 2) 1 Month Before the Pandemic: 54.3% Caucasian, 2.9% Black or African American, 34.3% Asian, and 8.6% declined to answer; 3) During the Pandemic: 42.4% Caucasian, 39.4% Asian, and 18.2% declined to answer. A proxy of socioeconomic status was estimated using the average (for the subject's mother and father) years of parental education. Depression was measured using Beck Depression Inventory (Beck et al., 1961). State and trait anxiety was measured using the Spielberger State and Trait Anxiety Inventory (Spielberger et al., 1983). Subclinical psychotic symptoms, or psychotic experiences (PEs), were measured using the Peters Delusion Inventory (Peters et al., 1999). There were no significant differences in demographic variables ($p > .056$) across groups. State anxiety was significantly higher in the group assessed during the pandemic compared to the group assessed >6 months before the pandemic ($t(190) = 1.987$, $p = .048$). There were no other significant differences in symptom levels across the three groups (all $p > .066$).

	Baseline (n=19)	Before the Pandemic (n=12)	During the Pandemic (n=12)
Age	30.1 (11.3)	33.0 (11.1)	33.3 (11.2)
Gender	45.0%	41.7%	41.7%
Ethnicity	30.0%	33.3%	33.3%
SES	15.1 (2.7)	14.7 (3.1)	14.7 (3.1)
Depression	2.0 (3.9)	1 (2.6)	2 (3.8)
State Anxiety	29.0 (9.0)	27 (9.0)	29 (7.9)
Trait Anxiety	31.0 (10.4)	31 (12.2)	34 (10.3)
PEs	1.0 (1.8)	1.0 (1.4)	2.0 (1.7)

Table S2. Demographic and psychological characteristics of Cohort 2. Mean (mean (standard deviation)) age (in years), socioeconomic status (SES), psychopathology levels, as well as gender (% female) and ethnicity (% Hispanic) are shown for Cohort 2 at baseline (the full cohort), before the pandemic (i.e., the subset of the baseline group who were later assessed during the pandemic) and during the pandemic. The racial composition of the Cohort 2 groups was as follows: 1) Baseline: 73.7% Caucasian, 10.5% Black or African American, and 15.8% Asian; 2) Before/During the Pandemic: 66.7% Caucasian, 16.7% Black or African American, and 16.7% Asian. A proxy of socioeconomic status was estimated using the average (for the subject's mother and father) years of parental education. Depression was measured using the Beck Depression Inventory (Beck et al., 1961). State and trait anxiety was measured using the Spielberger State and Trait Anxiety Inventory (Spielberger et al., 1983). Subclinical psychotic symptoms, or psychotic experiences (PEs), was measured using the Peters Delusion Inventory (Peters et al., 1999). The Before and During groups are the identical subset of subjects of the Baseline sample. There were no significant changes in symptom measures between the Before and During time points (all $p > .126$).

(A)

3-Way Repeated Measures ANOVA (Time*Modality*SDP Type) Results	*F*	*p*
Time	17.479	0.002
Modality	3.042	0.109
SDP Type	41.774	< 0.001
Time * Modality	5.704	0.036
Time * SDP Type	3.55	0.086
Modality * SDP Type	0.615	0.45
Time * Modality * SDP Type	0.058	0.813

(B)

T-Test Results, Before vs. During the Pandemic	*t*	*p*
Real Passive	-5.732	<0.001
Real Active	-3.863	0.003
Virtual Passive	-2.918	0.014
Virtual Active	-3.082	0.01

(C)

T-Test Results, Passive vs. Active	*t*	*p*
Before Real	4.25	0.001
Before Virtual	3.526	0.005
During Real	6.563	<0.001
During Virtual	4.634	0.001

Table S3. ANOVA and t-test results: comparisons of personal space size in response to real and virtual humans before versus during the pandemic in Cohort 2. (A) A 3- way repeated measures ANOVA (Time*Modality*Stop Distance Procedure (SDP) Type) conducted with the personal space size (D1) data shows a significant main effect of time, a significant main effect of SDP type (passive vs. active trials) and a significant interaction between time (Before vs. During the Pandemic) and modality (Real vs. Virtual). **(B)** Post-hoc t-tests reveal that the main effect of time was due to significant increases in personal space size during (versus before) the COVID-19 pandemic across all four measurements. **(C)** Post-hoc t-tests also revealed that the main effect of SDP type arose from significantly higher personal space size measurements for the passive, compared to the active, SDP trials across both modalities and time points, consistent with the findings of prior studies. The interaction between time and modality was due to slightly (Passive: 14.4cm, Active: 10.0cm) higher SDP values for the virtual, compared to the real, measurements at baseline (Passive: $t(11) = 2.550$, $p = .027$; Active: $t(11) = 3.968$, $p = .002$).

References for Supplementary Materials

Beck, A.T., Ward, C.H., Mendelson, M., Mock, J., and Erbaugh, J. (1961). An inventory for measuring depression. *Arch Gen Psychiatry* 4**,** 561-571. doi: 10.1001/archpsyc.1961.01710120031004.

Bruck, S., and Watters, P. (2009). Cybersickness and Anxiety During Simulated Motion: Implications for VRET. *Stud Health Technol Inform* 144**,** 169-173.

Burke, A.S., Shapero, B.G., Pelletier-Baldelli, A., Deng, W.Y., Nyer, M.B., Leathem, L., et al. (2019). Rationale, Methods, Feasibility, and Preliminary Outcomes of a Transdiagnostic Prevention Program for At-Risk College Students. *Front Psychiatry* 10**,** 1030. doi: 10.3389/fpsyt.2019.01030.

DeCross, S.N., Farabaugh, A.H., Holmes, A.J., Ward, M., Boeke, E.A., Wolthusen, R.P.F., et al. (2020). Increased amygdala-visual cortex connectivity in youth with persecutory ideation. *Psychol Med* 50(2)**,** 273-283. doi: 10.1017/s0033291718004221.

DeTore, N.R., Luther, L., Deng, W., Zimmerman, J., Leathem, L., Burke, A.S., et al. (2022). Efficacy of a transdiagnostic, prevention-focused program for at-risk young adults: a waitlist-controlled trial. *Psychological Medicine***,** 1-10. doi: 10.1017/S0033291722000046.

Peters, E.R., Joseph, S.A., and Garety, P.A. (1999). Measurement of delusional ideation in the normal population: introducing the PDI (Peters et al. Delusions Inventory). *Schizophr Bull* 25(3)**,** 553-576. doi: 10.1093/oxfordjournals.schbul.a033401.

Sheehan, D.V., Lecrubier, Y., Harnett Sheehan, K., Janavs, J., Weiller, E., Keskiner, A., et al. (1997). The validity of the Mini International Neuropsychiatric Interview (MINI) according to the SCID-P and its reliability. *European Psychiatry* 12(5)**,** 232-241. doi: 10.1016/S0924-9338(97)83297-X.

Spielberger, C., Gorsuch, R., Lushene, R., Vagg, P.R., and Jacobs, G. (1983). *Manual for the State-Trait Anxiety Inventory (Form Y1 – Y2).*
